# Zebra finch (*Taeniopygia guttata*) shift toward aerodynamically efficient flight kinematics in response to an artificial load

**DOI:** 10.1242/bio.042572

**Published:** 2019-05-29

**Authors:** Anthony B. Lapsansky, Jennifer A. Igo, Bret W. Tobalske

**Affiliations:** 1 Field Research Station at Fort Missoula, Division of Biological Sciences, University of Montana, Missoula, MT 59812, USA; 2 University College Cork, School of Biological, Earth & Environmental Sciences, Distillery Fields, North Mall, Cork, T23 N73K Ireland

**Keywords:** Intermittent flight, Flap-bounding, Zebra finch, Kinematics

## Abstract

We investigated the effect of an added mass emulating a transmitter on the flight kinematics of zebra finches (*Taeniopygia guttata*), both to identify proximal effects of loading and to test fundamental questions regarding the intermittent flight of this species. Zebra finch, along with many species of relatively small birds, exhibit flap-bounding, wherein the bird alternates periods of flapping with flexed-wing bounds. Mathematical modeling suggests that flap-bounding is less aerodynamically efficient than continuous flapping, except in limited circumstances. This has prompted the introduction of two major hypotheses for flap-bounding – the ‘fixed-gear’ and ‘cost of muscle activation/deactivation’ hypotheses – based on intrinsic properties of muscle. We equipped zebra finches flying at 10 m s^−1^ with a transmitter-like load to determine if their response was consistent with the predictions of these hypotheses. Loading caused finches to diverge significantly from their unloaded wingbeat kinematics. Researchers should carefully consider whether these effects impact traits of interest when planning telemetry studies to ensure that tagged individuals can reasonably be considered representative of the overall population. In response to loading, average wingbeat amplitude and angular velocity decreased, inconsistent with the predictions of the fixed-gear hypothesis. If we assume that finches maintained muscular efficiency, the reduction in amplitude is inconsistent with the cost of the muscle activation/deactivation hypothesis. However, we interpret the reduction in wingbeat amplitude and increase in the proportion of time spent flapping as evidence that loaded finches opted to increase their aerodynamic efficiency – a response which is consistent with the latter hypothesis.

## Abstract (precis)

**Summary:** When equipped with an artificial load, zebra finch diverge from their typical flap-bounding pattern of flight, potentially reducing their muscular efficiency, and adopt more aerodynamically efficient kinematics.

## INTRODUCTION

Telemetry is a widely used technology for tracking the movement of animals and has expanded our knowledge of countless species and systems (Hussey et al., 2015; Kays et al., 2015). But as we enter the ‘golden age of animal tracking science’ (Kays et al., 2015) there is increased pressure to understand the impact of telemetry on study animals (Mcintyre, 2015). The animal must carry a transmitter for the researcher to pinpoint that animal's position, and this is generally considered a burden (Barron et al., 2010; Calvo and Furness, 1992; Murray and Fuller, 2000). Potential negative effects of telemetry include an increase in the cost of locomotion due to the mass of – or drag on – the transmitter, a reduction in maneuverability, an increase in conspicuousness, a reduction in the effectiveness of insulation, or behavior modification.

The question of how telemetry affects study animals is pertinent from both an animal-welfare and statistical perspective. Researchers wish to avoid inadvertently harming their study organisms, but even when the effects are slight, telemetry has the potential to bias data. A key assumption of telemetry studies is that tagged animals are representative of the overall population (i.e. a representative sample), but if the transmitter significantly affects the behavior of tagged animals, then this assumption is clearly violated (Guthery and Lusk, 2004; Millspaugh and Marzluff, 2001).

We investigated the impact of the added mass of a transmitter on the flight kinematics of zebra finches (*Taeniopygia guttata*), both to identify proximal effects of loading and as a means to investigate fundamental questions regarding the flying gait of this species. The zebra finch is a species of small passerine that uses a common form of intermittent flight known as ‘flap-bounding’, in which the bird alternates flapping phases with flexed-wing bounds (Rayner, 1985; Tobalske, 2001; Usherwood, 2016).

Flap-bounding is exhibited by diverse species of relatively small birds, including most passerines, woodpeckers and some smaller owls, yet mathematical modeling of bird flight suggests that this flight style requires a higher aerodynamic power output than continuous flapping across all conditions, except perhaps at high flights speeds (Rayner, 1985; Ward-Smith, 1984a,b), or when flying fast into a headwind (Sachs, 2013; Sachs and Lenz, 2011). Regardless, many flap-bounding species continue to bound in slow and hovering flight.

If flap-bounding requires a higher aerodynamic power output, then this gait should also increase a bird's cost of transport – the amount of energy required to travel a unit distance – relative to continuous flapping (Schmidt-Nielsen, 1972). Thus, it is surprising that many species exhibit flap-bounding during migration (Rayner, 1985) when they are expected to fly efficiently to conserve limited energy stores (Vincze et al., 2018).

There are two major hypotheses that attempt to explain the use of flap-bounding by small birds under circumstances that are not aerodynamically advantageous. The fixed-gear hypothesis posits that the muscles of small birds have been ‘tuned’ for high-powered flight via adaptive evolution (Rayner, 1985). As described, birds require the capacity for high-powered flight as it allows them to take off from the ground and escape predators through rapid acceleration. But because muscles of a given fiber type are most efficient over a narrow range of contractile velocities (Hill, 1950), and small birds may only have space in their pectoralis (the primary downstroke muscle in birds) for one fiber type, it behooves these species to maintain constant contractile velocities. This allows for greater mechanical work production per unit of ATP spent in contracting a given muscle fiber, ignoring the cost of activating and deactivating the muscle. Thus, small birds should only exhibit wingbeats which produce high aerodynamic power. When high power output is not required, such as at intermediate flight speeds, birds bound periodically to maintain speed rather than contract their muscles at inefficient velocities. The fixed-gear hypothesis further predicts that wing design (generally rounded, low aspect ratio) restricts flap-bounding birds to near-constant kinematics across flight speeds. Thus, birds alter only the proportion of time they spend flapping to vary their aerodynamic power output. The proportion of time that a bird spends flapping relative to its total flight time, i.e. its ‘flapping ratio’, was first symbolized as *a* (Rayner, 1985); this denotation will be continued here. Since its introduction, empirical studies have not provided support for the fixed-gear hypothesis (Ellerby and Askew, 2007; Tobalske, 1994, 2005; Tobalske et al., 1999), but the idea remains pervasive in popular culture (e.g. Wikipedia Contributors, 2017).

Recently, a second hypothesis was presented for the existence of flap-bounding, here referred to as the cost of muscle activation/deactivation hypothesis (Usherwood, 2016). Briefly summarized, small birds must beat their wings at a high angular velocity in order to produce sufficient thrust to overcome drag and thus orient their net force production forward. Using such quick downstrokes requires a high energy output over a short time interval – i.e. high power – and thus requires activating a large volume of muscle. Muscle activation and deactivation are metabolically costly; therefore, to get the most mechanical work out of activating that volume of muscle, a long duration downstroke is favorable. The combination of these two pressures on the kinematics of the downstroke – high velocity and long duration – favors a high amplitude wingbeat, resulting in an overall high aerodynamic power output. Thus, these birds produce excess aerodynamic power at most flight speeds and periodically bound to avoid acceleration. As in the fixed-gear hypothesis, by increasing the efficiency of the flight muscles, flap-bounding is more efficient overall, despite the reduction in aerodynamic efficiency. We refer to mechanisms which increase or decrease mechanical work production given the cost of muscle activation and deactivation as affecting the ‘activation/deactivation efficiency’ of muscle. This nomenclature is used to distinguish this pressure with the ‘contractile efficiency’ of the fixed-gear hypothesis, which does not consider the cost of activating and deactivating muscle.

It should be noted that, unlike the fixed-gear hypothesis, the cost of muscle activation/deactivation hypothesis does not consider the muscles of flap-bounding birds to be locked into a specific set of (muscularly efficient) contractile parameters. Rather, the latter hypothesis views flap-bounding as a response, in real-time, to a muscular versus aerodynamic efficiency tradeoff (Usherwood, 2016). This means that while a finch is presumed to compromise aerodynamic efficiency in favor of activation/deactivation efficiency under native conditions, it may compromise activation/deactivation efficiency in favor of aerodynamic efficiency under other conditions (e.g. when loaded).

The addition of a load such as a transmitter increases the power required for flight at a given velocity due to an increase in the force the bird must output to counteract gravity and the drag on the load. Thus, the bird must increase its both its vertical (weight-support) and horizontal (thrust) force production to compensate for the load and maintain elevation.

To compensate for the power requirement of the load while achieving economical flight, finches have two options (Table 1). First, they could maintain or perhaps increase the efficiency of their muscle use. If finches are governed by the fixed-gear hypothesis, compensating for the load while maintaining contractile efficiency is the only option. To do this, finches should exhibit constant downstroke velocities (via constant contractile velocities) and simply increase their flapping ratio to compensate for the power required by the load (Rayner, 1985). If governed by the cost of muscle activation/deactivation hypothesis (Usherwood, 2016), compensating for the load while maintaining activation/deactivation efficiency would mean finches should exhibit constant downstroke duration and wingbeat amplitude. They should also increase their flapping ratio to counteract the mass of the load. An increase in activation/deactivation efficiency could occur by increasing the duration of the downstroke, either by increasing wingbeat amplitude or decreasing downstroke velocity (Usherwood, 2016). Second, finches could opt to increase their aerodynamic efficiency (meaning that they could decrease the power output required to fly at a given speed) by increasing their flapping ratio and decreasing wingbeat amplitude. These two responses would theoretically create a steadier momentum jet with less induced power cost (Muijres et al., 2011; Rayner, 1985; Sachs and Lenz, 2011; Usherwood, 2016; Ward-Smith, 1984b) and the increase in flapping ratio would compensate for the added power-cost incurred by loading. This response should not be possible for finches governed by the fixed-gear hypothesis (Rayner, 1985), but could occur via a reduction in activation/deactivation efficiency (because a shallower wingbeat means a shorter downstroke duration and thus less work is produced for activating that volume of muscle) for finches governed by the cost of muscle activation/deactivation. Note that all three options in Table 1 predict an increase in flapping ratio in response to loading, and that these two options are not mutually exclusive.

**Table 1.**
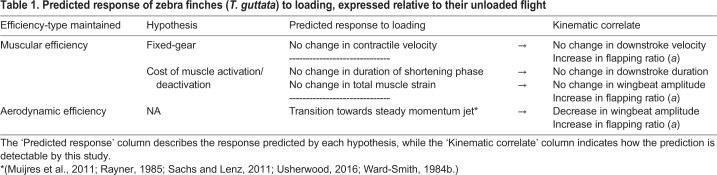
**Predicted response of zebra finches (*T. guttata*) to loading, expressed relative to their unloaded flight**

Previous studies, which have allowed birds to select their own flight speed, document that the animals opt for lower flight velocities when equipped with an artificial load (Gessaman and Nagy, 1988; Hughes and Rayner, 1991; Nudds and Bryant, 2002; Videler et al., 1988) except at very high loads (Hambly et al., 2004). We chose to enforce a flight speed of 10 m s^−1^ for both unloaded and loaded flights, rather than confound the variables of loading and flight speed, and use the effects of loading to test the predictions of the fixed-gear and cost of muscle activation/deactivation hypotheses.

## RESULTS

Finches significantly increased their flapping ratios in response to loading (*P*=0.043, *n*=5, Table 2) from an unloaded average of 0.67±0.07 to 0.79±0.06 under load. There was no effect of loading on wingbeat frequency (*P*=0.674, *n*=5, Table 3).

**Table 2.**
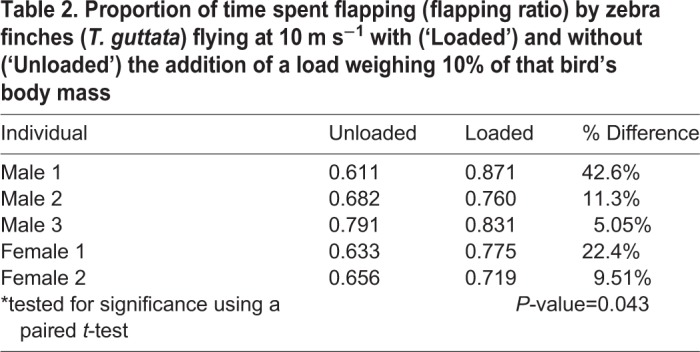
**Proportion of time spent flapping (flapping ratio) by zebra finches (*T. guttata*) flying at 10 m s^−1^ with (‘Loaded’) and without (‘Unloaded’) the addition of a load weighing 10% of that bird's body mass**

**Table 3.**
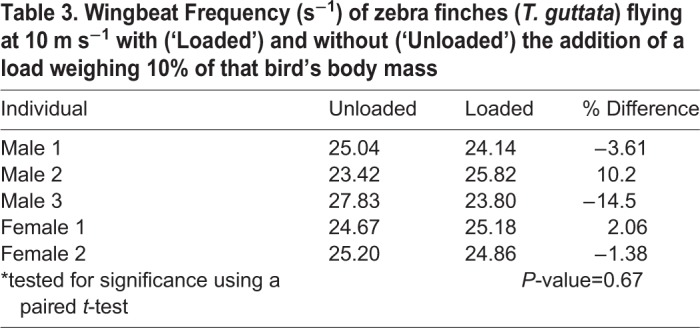
**Wingbeat Frequency (s^−1^) of zebra finches (*T. guttata*) flying at 10 m s^−1^ with (‘Loaded’) and without (‘Unloaded’) the addition of a load weighing 10% of that bird's body mass**

**Fig. 1.**
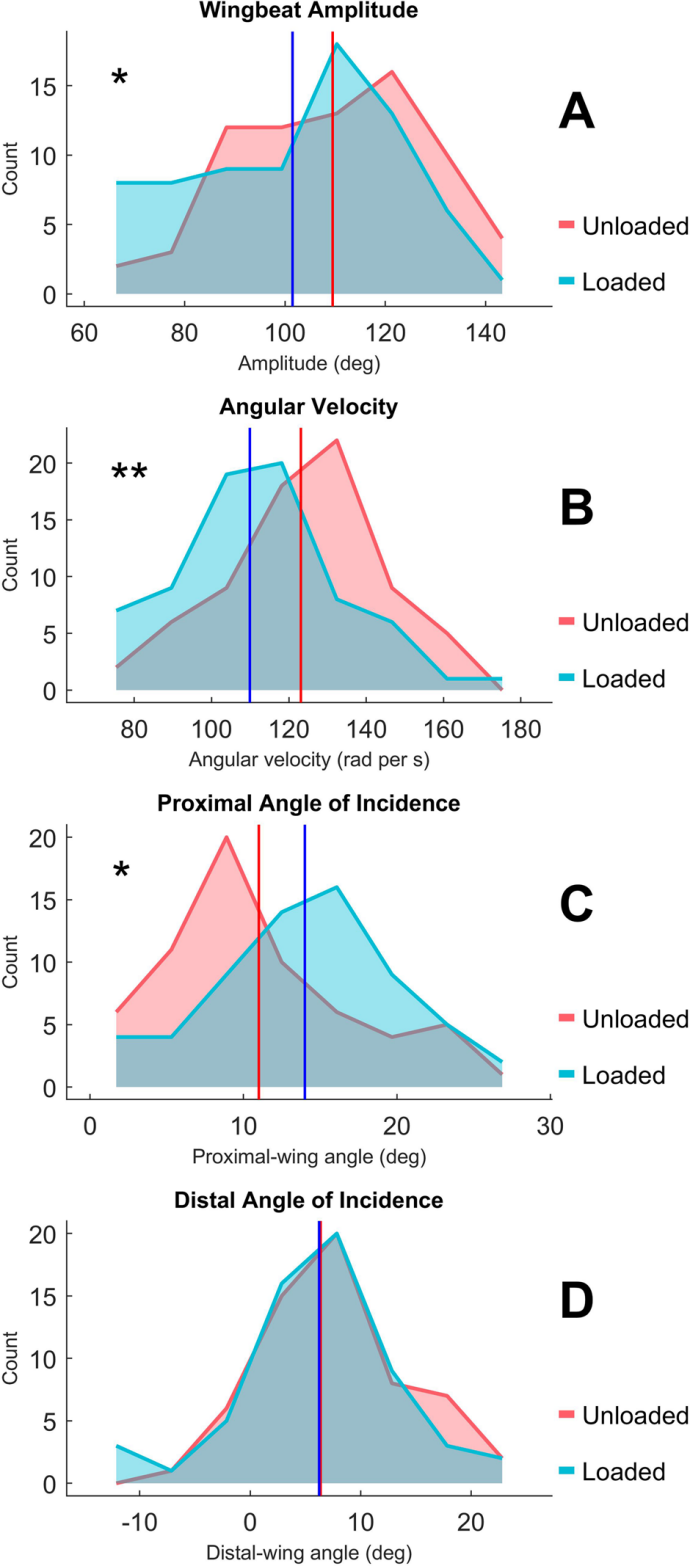
**Kinematic parameters describing the wingbeats of zebra finch (*T. guttata*) when equipped with a load weighing 10% of their body mass (‘Loaded’) versus the kinematics of wingbeats when those same birds were unweighted (‘Unloaded’).** Each panel displays the distribution of parameter values measured across all individuals (*n*=5) with the unweighted average for each condition indicated by a vertical line of the same color. Linear mixed-effects models for each parameter were tested for a significant effect of loading using the KRmodcomp function in the pbkrtest package in R. (A) Loaded wingbeats were of significantly reduced amplitude (*P<0.01, *n*=144) relative to loaded wingbeats, from an unloaded average of 109.5±19.5° to 101.5±20.7° under load. (B) Loaded wingbeats were of significantly reduced downstroke velocity (**P<0.001, *n*=142), from an unloaded average of 123.1±21.0 rad s^−1^ to 109.9±21.6 rad s^−1^ under load. (C) Loaded wingbeats were of significantly increased angle of incidence (*P<0.01, *n*=126), from an unloaded average of 11.0±6.4° to 14.0±6.2° under load. (D) There was no significant effect of loading on distal angle of incidence (*P*=0.056, *n*=118).

Loaded wingbeats were of significantly reduced amplitude (*P*<0.01, *n*=144) relative to loaded wingbeats. The average amplitude of wingbeats sampled was reduced from an unloaded average of 109.5±19.5° to 101.5±20.7° under load (Fig. 1A). Further, loaded wingbeats were of significantly reduced downstroke velocity (*P*<0.001, *n*=142), from an unloaded average of 123.1±21.0 rad s^−1^ to 109.9±21.6 rad s^−1^ under load (Fig. 1B). The combination of these two responses meant that there was no significant change in downstroke duration of wingbeats (*P*=0.36, *n*=142), which averaged 0.016±0.003 s for unloaded wingbeats and 0.016±0.003 s for loaded wingbeats. Loaded wingbeats were of significantly increased proximal angle of incidence (*P*<0.01, *n*=126), from an unloaded average of 11.0±6.4° to 14.0±6.2° under load (Fig. 1C). There was no significant effect of loading on distal angle of incidence (*P*=0.056, *n*=118, Fig. 1D). Distal angle of incidence averaged 8.02±6.68° for unloaded wingbeats and 5.86±7.05° for loaded wingbeats. Occasionally, we computed wingbeats as having negative angles of incidence. This may have been caused by the simplified manner in which we calculated induced velocity, which averaged 1.14 m s^−1^ for unloaded wingbeats and 0.79 m s^−1^ for loaded wingbeats.

**Fig. 2.**
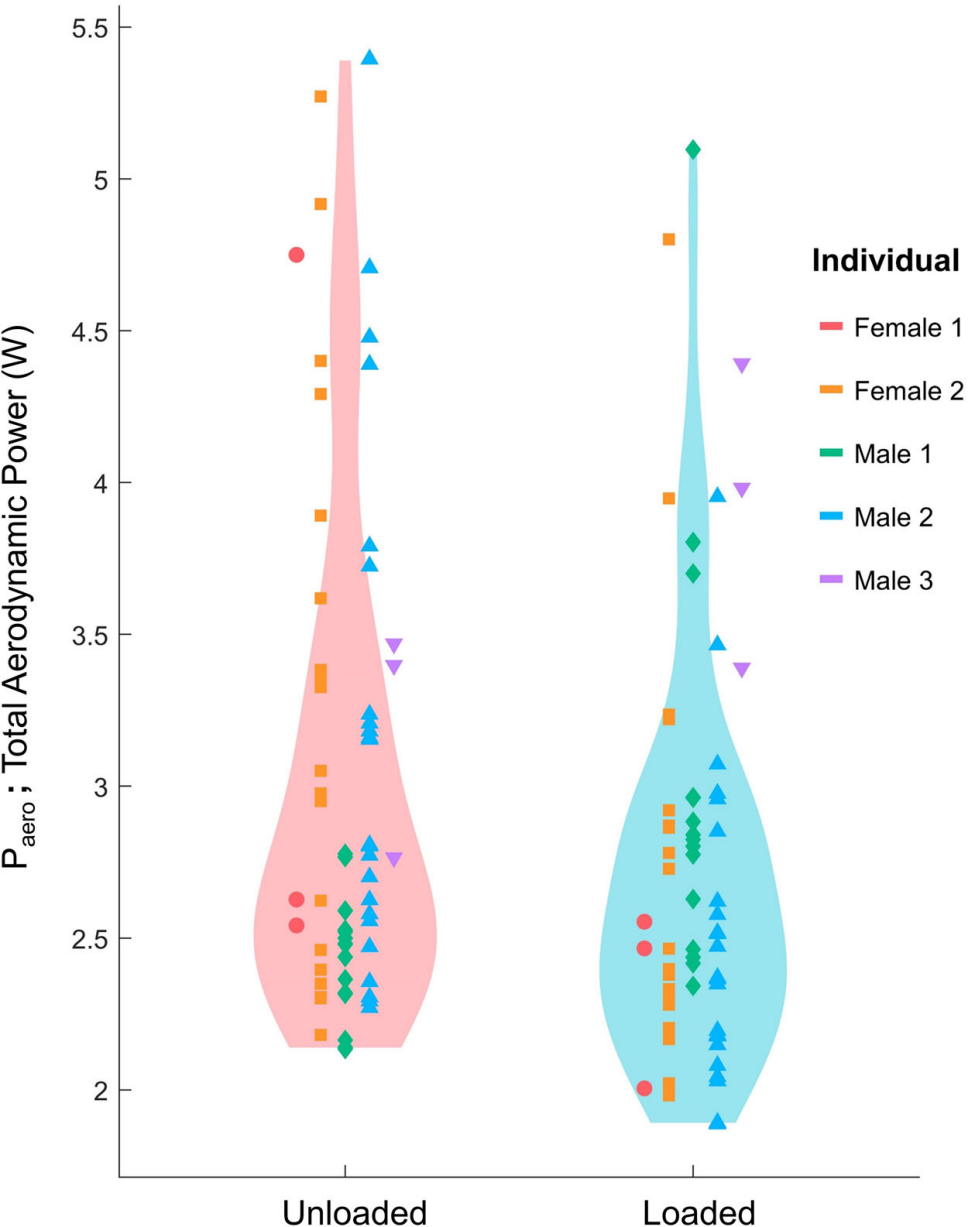
**Birds equipped with a payload weighing 10% of their body mass exhibited wingbeats (*n*=128) of significantly reduced total aerodynamic power, P_aero_, relative to their unloaded wingbeats (*P*=0.031; linear mixed-effects model, Kenward-Rogers-adjusted *P*-value).** The color/marker of each point indicates the individual finch from which that wingbeat was sampled, while the violins represent the distribution of the pooled wingbeats in each treatment. Average *P_aero_* was reduced from 3.03±0.82 W when unloaded to 2.74±0.69 W under load. The scatter of the points within each individual highlight that the wingbeats of zebra finch cannot be considered ‘fixed’, contrary to the prediction of the fixed-gear hypothesis.

The combination of reduced amplitude and downstroke velocity meant that wingbeats exhibited under loaded conditions were significantly reduced in *P_pro_* (*P*<0.001, *n*=128) – from 2.19±0.19 W when unloaded to 2.09±0.21 W under load – and, therefore, *P_aero_* (*P*=0.031, *n*=128) – from 3.03±0.82 W when unloaded to 2.74±0.69 W under load (Fig. 2). There was no significant difference in *P_ind_* (*P*=0.15, *n*=128), which averaged 0.786±0.792 W for unloaded wingbeats compared to 0.605±0.648 W under load.

## DISCUSSION

The significant effects of loading upon flapping ratio and a variety of wingbeat kinematics (Table 2, Figs 1A–C and 2) have important implications for telemetry studies. The mass of a transmitter has the potential to void the assumption that the kinematics and power output of tagged animals are representative of the population from which they are drawn. One caution to bear in mind is our added mass was 10% of body mass, double the informal ‘5%’ rule. However, recent meta-analysis indicates broadly negative effects of transmitters even when this rule is being followed (Barron et al., 2010). As well, the 5% rule has little empirical basis (Barron et al., 2010; except see Aldridge and Brigham, 1988), and instead appears to be based on an anecdote from an early publication (Brander and Cochran, 1969).

By altering the intermittent flight style of flap-bounding birds away from the norm, a transmitter has the potential to make a tagged individual the preferred target for predators, as predators often preferentially select abnormal individuals from a group of prey (Landeau and Terborgh, 1986; Mueller, 1975; Rutz, 2012; Theodorakis, 1989). Furthermore, by causing an increase in flapping ratio towards continuous flapping (Table 2), the addition of a transmitter may place a bird closer to its maximum aerodynamic power output and therefore limit its ability to accelerate in flight, gain altitude, and carry food. An animal's burst performance margin – the difference between the power an animal requires to perform routine behaviors and its maximum power output – is thought to be correlated with competitive ability, with individuals with narrower margins being poorer competitors (Altshuler, 2006; Altshuler et al., 2004; Skandalis et al., 2017). Therefore, if wild birds utilize an increase in flapping ratio to increase their aerodynamic power output, as demonstrated in this study, tagged birds may be less capable of accelerating away from predators or performing power-intensive activities. The impact of either of these behavioral modifications will likely lower the fitness of tagged birds, either by increasing mortality or by decreasing the ability to acquire mates or defend resources. These effects of tagging are well-documented, though the underlying cause is not always known (Mcintyre, 2015).

Furthermore, the impact of telemetry on an animal's burst performance margin may vary with the size of the animal, with larger flap-bounding species experiencing a more negative effect. Flapping ratio increases with body mass, which is interpreted as a negative scaling of the marginal power available for bounds as size increases (Tobalske, 1996, 2001). For example, budgerigars (*Melopsittacus undulatus*), which weigh approximately 2.5 times more than the zebra finches (Dunning, 2008), exhibit flapping ratios around 0.85 (Tobalske and Dial, 1994) compared to an average of 0.675 observed for unloaded zebra finches in this study. Flapping ratio also shows a positive relationship with increasing mass among 12 passerine and nine woodpecker species in the wild (Tobalske, 2001). As larger species exhibit higher flapping ratios, they likely have lower burst performance margins than smaller species (Skandalis et al., 2017) and would presumably be more negatively impacted by loading. Thus, increasing flapping ratio to compensate for the power cost of a load may only be possible with species with relatively low flapping ratios, like zebra finch.

The birds used in this study did not respond as predicted by the fixed-gear hypothesis. Loading caused a significant increase in flapping ratio (Table 2), as expected under the fixed-gear (Rayner, 1985) hypothesis; however, the observed changes in amplitude, proximal angle of incidence and, importantly, downstroke velocity are inconsistent with the expectations of the fixed-gear hypothesis. The fixed-gear hypothesis posits that small birds are constrained to exhibit only high-powered wingbeats, and therefore should not vary the kinematics of their wingbeats due to loading. Each wingbeat should output constant power, regardless of whether the bird is loaded, and birds should compensate for loading simply by increasing flapping ratio. The significant effects of loading on wingbeat amplitude (Fig. 1A), downstroke velocity (Fig. 1B) and proximal angle of incidence (Fig. 1C) indicate that the kinematics of the wingbeat were not fixed, and that loaded wingbeats were significantly less powerful than unloaded wingbeats indicates that per-wingbeat-power is variable (Fig. 2). This is further reinforced when recognizing the significant amount of intra-individual variation in P_aero_, which alone highlights that the wingbeat kinematics of zebra finch should not be considered fixed, contrary to the prediction of the fixed-gear hypothesis.

Our failure to support the key prediction of the fixed-gear hypothesis – that muscle contractile velocity is constant – is consistent with past research. Tobalske and Dial (1994) found that budgerigars exhibit variation in muscle activity and wingbeat frequency across different flight speeds. Further work demonstrated variation with flight speed in downstroke velocity (Tobalske et al., 1999) and in muscle contractile velocity (Tobalske, 2005) in zebra finches and, separately, in zebra finches and budgerigars (Ellerby and Askew, 2007). However, the fixed-gear hypothesis had yet to be tested using a discrete alteration in the power-cost of flight, such as by the addition of a load, despite the original author's recommendation of said test (Rayner, 1985). Previous research has focused on variation in flight speed as a means to alter the power-cost of flight in a continuous manner.

The cost of muscle activation/deactivation hypothesis posits that flap-bounding results from the combination of the high metabolic cost of muscle activation and deactivation – which favors a long duration downstroke – and the need for small birds to beat their wings quickly to orient their net aerodynamic force forward. According to Usherwood (2016), the combination of these two constraints – long duration but high-velocity downstrokes – favors the use of a high amplitude wingbeat across all flight speeds. However, because drag imposes the constraint for a fast wingbeat, Usherwood (2016) notes that birds should increase downstroke velocity with increasing drag on the body. This can occur either due to increases in flight velocity or due to added parasite drag.

If birds elected to maintain activation/deactivation efficiency between unloaded and loaded treatments, the cost of muscle activation/deactivation hypothesis predicts that birds would maintain a high amplitude wingbeat to maximize the duration of their downstroke and increase their flapping ratio to compensate for the mass of the load. In addition to mass, a load also adds some amount of parasite drag to the bird, and thus we might expect to see an increase in downstroke velocity as well. In contrast to these predictions, loaded birds in our experiment exhibited wingbeats of significantly decreased amplitude (Fig. 1A) and downstroke velocity (Fig. 1B) relative to their unloaded wingbeats.

This suggests that loaded birds elected instead to increase the aerodynamic efficiency of their flight. By decreasing the amplitude of their wingbeats and increasing flapping ratio, a loaded bird produces a more ideal, steady momentum jet (Usherwood, 2016). This should increase the aerodynamic efficiency of their loaded flight relative to their unloaded flight (Usherwood, 2016; Usherwood et al., 2011). Unfortunately, our results neither support nor undermine the cost of muscle activation/deactivation hypothesis, as it considers flap-bounding to be a plastic response to a trade-off between muscular and aerodynamic efficiency. In other words, it is conceivable that a loaded bird operating under the cost of muscle activation/deactivation hypothesis might achieve greater efficiency overall by reducing the muscular efficiency of its flight muscles in exchange for an increase in aerodynamic efficiency. This would explain the reduction in wingbeat amplitude (Fig. 1A) and increase in flapping ratio (Table 2) that we detected, though the latter does not necessitate a decrease in activation/deactivation efficiency. Alternatively, if the cost of muscle activation/deactivation hypothesis does not explain flap-bounding, it is likewise conceivable that a bird might need to increase flapping ratio simply to accommodate the added mass of the load. The decrease in downstroke velocity and wingbeat amplitude could then be explained if the increase in flapping ratio, to meet the weight-support-demands caused by the mass of the load, more than met the thrust demands caused by drag on the load (Usherwood, personal communication).

Because we sampled wingbeats opportunistically, the distribution of kinematic data is not weighted evenly across our five individuals (e.g. Fig. 2). Thus, the significant effects of loading to wingbeat amplitude, downstroke velocity and proximal angle of incidence are likely driven by the responses of those individuals that were more heavily sampled. However, all five birds dramatically increased their flapping ratio, consistent with our interpretation that birds increased their aerodynamic efficiency in response to loading.

*Post hoc* analysis of previous research, in which birds were flown at varying speeds to alter the power cost of flight, is mixed with regards to the cost of muscle activation/deactivation hypothesis. Wingbeat amplitude does not vary significantly across flight speeds in starlings (Tobalske, 1995), as predicted, but varies significantly in zebra finches (Tobalske et al., 1999). Further, downstroke velocity, pectoralis strain and strain rate varies according to U-shaped curve with flight speed in zebra finches (Tobalske et al., 1999), rather than a simple increase with flight speed, and a similar result was obtained by recording strain rate in the pectoralis of budgerigars (Ellerby and Askew, 2007).

As predicted by both hypotheses (Rayner, 1985; Usherwood, 2016), finches manipulate flapping ratio as a mechanism to meet the power demands of loaded flight (Table 2). This is consistent with past work which studies birds across a range of flight speeds (Ellerby and Askew, 2007; Tobalske and Dial, 1994; Tobalske et al., 1999). Interestingly, while the birds in our study increased their flapping ratio by an average of 15.1% in response to loading, no bird adopted a continuous flapping gait. This would have maximized their aerodynamic efficiency if paired with wingbeats of shallow amplitude. This suggests either that some degree of flap-bounding is more efficient overall even under loaded conditions, perhaps due to the mechanism outlined by the cost of muscle activation/deactivation hypothesis, or that our finches were unable to flap continuously, perhaps due to the innervation of their flight muscles. That finches did not significantly vary their downstroke duration despite loading suggests further support for the cost of muscle activation/deactivation. However, this may due to the low resolution of this measurement. We marked downstrokes as beginning and ending at times corresponding to individual frames of video, meaning our data on downstroke duration is discrete and therefore coarse.

Because we chose to enforce a flight speed of 10 m s^−1^ for both unloaded and loaded flights, and because we studied birds in level flight rather than during takeoff, there are relatively few studies to which we can compare our results. This is because birds may modulate their speed or kinematics to maintain the power required for flight (Hambly et al., 2004; Nudds and Bryant, 2002). The exception seems to be one study which analyzed the banked turns of pigeons in free flight in which birds adopted a variety of flight speeds (Usherwood et al., 2011). Banked turning increased the induced power cost of flight not unlike adding a load, and Usherwood et al. (2011) found that pigeons decreased wingbeat amplitude and increased wingbeat frequency during banked turns. The former result was echoed by our study; however, we found no significant change in wingbeat frequency in response to loading.

Based on the predictions of the cost of muscle activation/deactivation hypotheses, our results suggest the finches favor aerodynamic efficiency in response to loading rather than activation/deactivation efficiency. Further, our results indicate that the fixed-gear hypothesis fails to explain flap-bounding in this species; however, there are three important caveats. First, referring specifically to the cost of muscle activation/deactivation hypothesis, we assume that birds in our experiment ‘attempted’ to fly efficiently, but this may not have been true. We attached loads to the finches immediately prior to recording their flight, and it is possible that they responded by disregarding efficiency altogether. We chose this method of weighting, rather than adding the load and then allowing the birds to acclimate, as an acclimation period would have allowed time for variation in the size and/or constituency of their flight muscles (Rayner, 1985). It is well-documented that individual birds can vary in body mass and composition on relatively short time scales due to natural factors (Landys-Ciannelli et al., 2003; Scott et al., 1994; Vézina and Williams, 2003) and artificial loading (Nudds and Bryant, 2002). In addition, our method more closely resembles the way birds are equipped with transmitters for research purposes (Bowlin et al., 2015; Şekercioğlu et al., 2015; Sjöberg et al., 2015). Whether the effects on wingbeat kinematics we observed are maintained in the long-term remains to be tested. Second, we assume that wingbeat kinematics (e.g. downstroke velocity) are directly proportional to the muscle contractile properties, such as contractile velocity. This appears to be a reasonable assumption given that studies of *in vivo* muscle strain and strain rate are comparable with estimates from kinematics (Biewener et al., 1998; Ellerby and Askew, 2007; Tobalske, 2005). Nevertheless, variation could exist between the timing of wing motion and the underlying muscle activity. Third, while both hypotheses are based on the idea that flap-bounding is more efficient for these species, we did not directly measure metabolism. Rather, we rely on the predicted manifestations of the fixed-gear and cost of muscle activation/deactivation hypotheses to test their proposed mechanism.

Future work should address these limitations, either by directly recording muscle cycling *in vivo*, by using cross-fostering to produce continuous flapping individuals from flap-bounding species, or by working in the field to increase the likelihood that birds exhibit efficient patterns of motion.

An ideal test of either hypothesis would be to train a flap-bounding species to switch on command between a flap-bounding and a continuous flapping gait. This would allow the researcher to use masked respirometry (Morris et al., 2010) to determine if flap-bounding is more efficient than continuous flapping for these species; an idea which, though it is the basis for both the fixed-gear and cost of muscle activation/deactivation hypotheses, remains unconfirmed. However, training birds in this manner would be extremely difficult.

Alternatively, one could isolate muscle fibers of the pectoralis from a flap-bounding species and impose cyclical contractions characteristic of flap-bounding and continuous flapping. By quantifying the heat production by the muscle fibers relative to their mechanical power output under both conditions, one could determine whether cycling patterns characteristic of flap-bounding confer an advantage for flap-bounding species despite the added aerodynamic cost (Barclay, 1994; Curtin and Woledge, 1993; Ellerby and Askew, 2007). If so, one would then need to devise further experiments using these isolated muscles to test the predictions and logic behind the fixed-gear and cost of muscle activation/deactivation hypotheses.

One could also attempt to produce continuous flapping individuals from flap-bounding species using cross-fostering. Cross-fostering – the raising of an animal by surrogate parents of another species – has been shown to impact movement patterns in birds (Moore, 1992), including flying gait (Rowley and Chapman, 1985). Thus, one could potentially raise zebra finch offspring by using continuous flapping surrogate-parents to produce continuous flapping zebra finches. The efficiency of these individuals could then be compared to finches raised by their own species using masked respirometry (Morris et al., 2010) to determine if flap-bounding offers an efficiency advantage over continuous flapping in this species. Limitations of this approach are that cross-fostering would likely affect more behavioral traits than just gait, and the likelihood of achieving a complete shift to continuous flapping is unknown.

A fourth approach would be to use kinematic data collected from wild, free-flying birds to test the predictions of both hypotheses relating to downstroke velocity. The fixed-gear hypothesis predicts that downstroke velocity should remain constant across all flight speeds for a given species, whereas the cost of muscle activation/deactivation hypothesis predicts that downstroke velocity should increase in proportion with body drag, which increases exponentially with flight speed. Performing these measurements in the field would presumably increase the likelihood that the animals would exhibit kinematic patterns which maximize their efficiency. However, working in the field would also greatly increase the difficulty of obtaining kinematic data.

During the review process, a reviewer suggested two future tests to increase understanding of flap-bounding and the impacts of telemetry on flight performance. First, to distinguish between the effects of the weight of the transmitter on wingbeat kinematics and those due to the dimensions of that transmitter, researchers could equip zebra finches with either a weighted load (as in this study) or a light ‘sham’ transmitter of the same dimensions. This experiment would increase our understanding of the impacts of telemetry and may add to our knowledge of flight in general (in isolation from telemetry). A similar experiment proved effective for understanding foot-propelled diving in cormorants (Ribak et al., 2006). Second, some flap-bounding species gain weight when gravid without notable changes to their flight muscle mass (e.g. Kullberg, 2002), though this is not the case for zebra finch (Houston et al., 1995). Thus, researchers could treat these species as ‘natural experiments’ to potentially uncover the mechanisms responsible for flap-bounding.

In conclusion, the addition of a transmitter-like load caused zebra finches to diverge significantly from their unloaded flight kinematics. Researchers should carefully consider whether these effects impact traits of interest when planning telemetry studies to ensure that tagged individuals can reasonably be considered representative of the overall population. We interpret that the finches opted to increase the aerodynamic efficiency of their flight by increasing the percentage of time spent flapping and by decreasing their wingbeat amplitude. The reduction in amplitude and downstroke velocity was not consistent with the predictions of the fixed-gear hypothesis. Future work should address the cost of muscle activation/deactivation hypothesis either in natural population or measure some aspect of metabolism directly.

## MATERIALS AND METHODS

### Birds and training

Zebra finches (*n*=5) were purchased from a commercial breeder as part of a separate research project and housed in an indoor aviary at the Field Research Station at Fort Missoula, Missoula MT, USA. Three individuals were male and two were female. All birds were approximately 1-year old at the time of the study. Birds were trained to fly in the flight chamber of a variable speed wind tunnel at 10 m s^−1^ following the methods described elsewhere (Tobalske et al., 1999). We report equivalent airspeed *V_e_* rather than true airspeed (Pennycuick et al., 1997). A thorough description of the wind tunnel can be found in the following source (Tobalske, 2005). The study was conducted in June of 2017. Neither female produced an egg during or immediately after the study. Thus, it is unlikely that either were gravid. Upon completion of this study, all birds were adopted out to be kept as pets. All care and procedures were approved by the University of Montana Institutional Animal Care and Use Committee (IACUC).

### Experimental procedure

The flight of each finch was recorded under both unloaded and loaded conditions with a 7-day interlude between treatments. The initial treatment a finch received was assigned randomly to avoid bias due to the timing of treatment. The load consisted of a non-toxic, tungsten fishing weight manually shaved down to 10% of a given individual's body mass, as measured on an electronic scale (ACCULAB model VI-1200, accurate to 0.01 g, <1% of body mass). The load was attached directly to the skin of the bird's back on the dorsal midline of the body and approximately halfway between the shoulders and the base of the tail using cyanoacrylate adhesive <1 min before their flight was recorded. This location was estimated by A.B.L. by eye based on A.B.L.’s knowledge of avian anatomy and experience in mounting telemetry devices to falconry birds. The location was chosen over other possibilities (e.g. leg mount) as it is likely to be dorsal to the bird's center of mass during flight (Tobalske, 2005). Using cyanoacrylate adhesive is a widely used method for attaching telemetry transmitters (Barron et al., 2010).

We chose to add a load weighing 10% of body mass to increase our ability to detect the effects of added mass on the wingbeat kinematics of flap-bounding zebra finch. While 10% is heavier than is typically recommended for the maximum weight of a transmitter, the often cited ‘5%’- or ‘3%’-rule of thumb has little empirical basis (Barron et al., 2010; except see Aldridge and Brigham, 1988), and instead appears to be based on an anecdote from an early publication (Brander and Cochran, 1969).

Prior to recording, we marked the following anatomical points on the left wing of each finch (along with the base of their tail) using non-toxic, acrylic paint: shoulder, wrist, 9th primary, 5th primary, 4th secondary. We recorded the flight of each finch under each condition using digital video from three positions. Two Photron 1024 PCI cameras (Photron USA Inc., San Diego, CA, USA) provided lateral and dorso-cranial views, respectively, while one Photron SA-3 (Photron USA Inc.) provided a dorso-caudal view. The three cameras were synchronized using a transistor-transistor logic (TTL)-pulse. The flight chamber was illuminated using three 650W halogen lights (Lowel Tota-light, Lowel-Light Manufacturing, Inc., Brooklyn, NY, USA). Finches were recorded at 250 Hz with a shutter speed of 1/5000s. Finches were recorded until the three cameras captured video of multiple flapping bouts and bounds in succession. This usually occurred <30 s after placing the finches in the flight chamber. After recording their flight, the load was removed from loaded finches using a drop of acetone to dissolve the glue.

### Kinematics

Kinematic analyses of wingbeats were reconstructed in MATLAB (2017, MathWorks, Inc., Natick, Massachusetts, USA) using a DLT conversion (Hedrick, 2008) with additional analyses performed in MATLAB and IGOR Pro. (v. 6.01, Wavemetrics, Inc., Beaverton, OR, USA).

Wingbeat frequency (Hz) was calculated as the number of complete wingbeats divided by the total duration of all recorded flapping bouts. A flapping bout was defined as including all wingbeats occurring between two sequential bounds. Flapping ratio was calculated as the total duration of flapping bouts over the duration of those flapping bouts plus the duration of an equal number of associated bounds.

Variables obtained from kinematic analyses were used in constructing quasi-steady aerodynamic models incorporating actuator-disc theory and strip analysis (Askew et al., 2001; Norberg, 1989; Rayner, 1978; Tobalske et al., 2003). Using bird-centered coordinates, we measured wingbeat amplitude (degrees) and angular velocity (degrees s^−1^) for each downstroke. Angle of incidence (degrees) – the angle that a plane of the wing made with the overall airflow, including that induced by the finch – was determined at mid-downstroke as follows: we first found the vector normal to the proximal wing section (plane projected by the shoulder, wrist and 4th secondary) and distal wing section (plane project by the wrist, 5th primary and 9th primary) using the cross product. We then calculated the overall direction of the incoming airflow by summing the vector components of the freestream flow, the velocity of that wing section, and the vertical component of induced velocity (*V_i_*) as calculated according to the Rankine-Froude momentum jet theory (Aldridge, 1986; Pennycuick, 1975).(1)
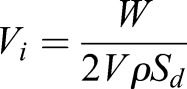
where W is body weight (including the load, if present), *ρ* is the density of the air (kg m−3) and *S_d_* is the horizontal disc area swept by the wings through global space (i.e. translating at 10 m s−1) for a given wingbeat, rather than a circle (Askew et al., 2001). Specifically, to calculate *S_d_* we used MATLAB to calculate the area of each trapezoid defined as the average distance in the y-direction between the wingtips at frames n and n+1 multiplied by the distanced traveled by the bird in the x-direction, accounting for the speed of freestream flow. We then used IGOR Pro to integrate the total area swept by the wings for each wingbeat by using local minima in the trapezoidal area as indicative of the end of a given wingbeat and start of the next. The angle of incidence for each wing section was found using the dot product formula for the angle between that normal to the wing section and that of the overall airflow at the time of mid-downstroke. The time of mid-downstroke was determined by using IGOR Pro to visually track the position of the wrist and locate the time of mid-downstroke for each wingbeat.

Average total aerodynamic power for each wingbeat (*P_aero_*) was calculated as the sum of induced *P_ind_*, profile *P_pro_*, and parasite *P_par_* power. Inertial power and the rate of change in kinetic and potential energy were ignored. Inertial power was ignored based on Norberg (1989), which states that inertial power costs may be neglected in fast and medium flight speeds. We assumed that changes in kinetic and potential energy to be negligible, as well, as birds flew at a constant 10 m s^−1^ and did not change elevation during analyzed sequences.

For the aerodynamic variables which contributed to *P_aero_*:(2)
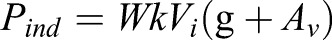
where *k* is the induced velocity correction factor (assumed to be 1.2), g is gravitational acceleration, and *A_v_* is vertical acceleration, tracked using the point on the base of the tail of the finch.(3)
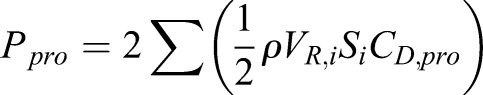
where *V_R,i_* is the resultant velocity of wing strip *i*, *S* is the surface area of a wing strip *i*, and *C_D,pro_* is the profile drag coefficient (assumed to be 0.02) (Norberg, 1989; Rayner, 1978). We assumed a constant value for *C_D,pro_* in consistency with the literature, but it should be mentioned that *C_D,pro_* may vary with lift (Klaassen van Oorschot et al., 2016). Strip analysis was performed by converting images of the left wing of each finch into a text document using ImageJ (v1.43u, National Institutes of Health, Bethesda, MA, USA). We then used a custom MATLAB script to slice each image into 15 strips and determine the area and average distance from the shoulder of each strip. The velocity of a given strip *V_R,i_* was taken as the product of the angular velocity of that downstroke and the average distance of that strip, *i*, from the shoulder. We assumed no change in average wing chord throughout the downstroke.(4)
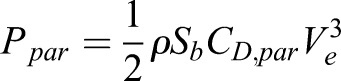
where *S_b_* is the projected equivalent flat-plate area of the body, calculated incorporating approximated body angle related to free-stream flow (Rayner, 1978), and *C_D,par_* is the parasite drag coefficient (assumed to be 0.13) (Askew et al., 2001; Rayner, 1999). We considered the body angle as relative to freestream flow, rather than incorporating the velocity induced by the bird, based on evidence that induced velocities are rather low near the body during flapping flight (Henningsson et al., 2014).

### Statistical analysis

We tested for a significant effect of loading on wingbeat frequency and flapping ratio using a paired *t*-test in Microsoft Excel (2016; Microsoft Corp., Redmond, WA, USA), considering each bird to have a single value under each condition.

For the kinematic parameters of wingbeat amplitude, downstroke velocity, proximal and distal angle of incidence, and each component of *P_aero_* we performed the following analysis: (1) we identified and removed outliers using the *isoutlier* (‘mean’) function in MATLAB. This procedure identifies elements of a series more than three standard deviations from the mean of that series. We removed outliers because digitization error can be compounded when taking the derivatives needed for computing velocities and accelerations. (2) Because we sampled opportunistically, we obtained data from an uneven number of wingbeats from each finch under the two conditions. To eliminate potential bias due to natural variation between finches, we randomly selected wingbeats from the condition with the greater sample size to obtain equal sample sizes for each finch. (3) Then, to test for a significant effect of loading, we employed a Linear Mixed-Effect Model with condition (i.e. with or without load, ‘Condition’) as a fixed effect and bird identity (*BirdID*) as a random effect in R version 3.31 (R Development Core Team 2016) using the pbkrtest package (Halekoh and Højsgaard, 2014). (4) Finally, we tested for an effect of Condition using the KRmodcomp function to test for a significant difference between models with and without the Condition effect (Faraway, 2016). This procedure uses the Kenward-Rogers adjusted *F*-test, which is a reliable method for determining *P*-values in Linear-Mixed Effects models (Luke, 2017). Analyses of kinematic parameters included measurements on 118 to 144 wingbeats sampled from five birds (i.e. *n*=118 to 144 measures from five birds).

We list the unweighted averages and their standard deviations in the results section for kinematic parameters. Note that the high standard deviations for the unweighted averages are due in part to the differences among individuals. The Linear Mixed-Effects model used to test for significant effects of loading on these parameters accounts for these intraspecific differences (Faraway, 2016; Halekoh and Højsgaard, 2014).

The raw digitized points for each bird and the data used for statistical testing are available on Figshare.
